# Sodium-glucose co-transporter-2 inhibitors reduce the risk of new-onset stroke in patients with type 2 diabetes: A population-based cohort study

**DOI:** 10.3389/fcvm.2022.966708

**Published:** 2022-08-09

**Authors:** Tsung-Kun Lin, Yong-Hsin Chen, Jing-Yang Huang, Pei-Lun Liao, Mei-Chun Chen, Lung-Fa Pan, Gwo-Ping Jong

**Affiliations:** ^1^Department of Pharmacy, Taoyuan Armed Forces General Hospital, Taoyuan, Taiwan; ^2^School of Pharmacy, National Defense Medical Center, Taipei City, Taiwan; ^3^Department of Public Health, Chung Shan Medical University, Taichung, Taiwan; ^4^Department of Occupational Safety and Health, Chung Shan Medical University Hospital, Taichung, Taiwan; ^5^Department of Medical Research, Chung Shan Medical University Hospital, Taichung, Taiwan; ^6^Institute of Medicine, Chung Shan Medical University, Taichung, Taiwan; ^7^Department of Medical Imaging and Radiological Science, Central Taiwan University of Science and Technology, Taichung, Taiwan; ^8^Department of Cardiology, Taichung Armed Forces General Hospital, Taichung, Taiwan; ^9^Department of Internal Medicine, Chung Shan Medical University Hospital and Chung Shan Medical University, Taichung, Taiwan

**Keywords:** new-onset stroke, SGLT2 inhibitor, type 2 DM, concurrent medication, ischemic stroke, hemorrhagic stroke

## Abstract

**Background:**

Epidemiological evidence suggests the association of diabetes with an increased risk of stroke. Clinical studies have investigated the effects of sodium-glucose co-transporter-2 (SGLT2) inhibitors on new-onset stroke (NOS), but the results are inconsistent.

**Objectives:**

To determine the association between the use of SGLT2 inhibitors and NOS in patients with type 2 diabetes mellitus (DM).

**Methods:**

We conducted a retrospective longitudinal cohort study based on the Taiwan Health Insurance Review and Assessment Service database (2016–2019). The primary outcome of the assessment was the risk of incident stroke by estimating hazard ratios (HRs) and 95% confidence intervals (CIs). Multiple Cox regression was applied to estimate the adjusted HR of NOS. Subgroup analysis was also conducted.

**Results:**

Among the 232,101 eligible patients with type 2 DM aged ≥ 20 years, SGLT2-inhibitor users were compared with non-SGLT2-inhibitor users based on age, sex, and the duration of type 2 DM matching at a ratio of 1:2. The event rate per 10 000 person-months was 9.20 (95% CI 8.95 to 9.45) for SGLT2-inhibitor users and 10.5(10.3–10.6) for non-SGLT2-inhibitor users. There was a decreased risk of NOS for SGLT2-inhibitor users (adjusted HR 0.85, 95% CI 0.82–0.88) compared with non-SGLT2-inhibitor users. Results for the propensity score-matched analyses showed similar results (adjusted HR 0.87, 95% CI 0.84–0.91 for both SGLT2-inhibitor users and non-SGLT2-inhibitor users).

**Conclusion:**

The risk of developing NOS was lower in patients with SGLT2-inhibitor users than in non-SGLT2-inhibitor users. The decreased risk of NOS in patients with type 2 DM was greater among patients with concurrent use of statins, biguanides, thiazolidinediones, and glucagon-like peptide-1 receptor agonists. We, therefore, suggest that the long-term use of SGLT2 inhibitors may help reduce the incidence of NOS in patients with type 2 DM.

## Introduction

The global incidence and prevalence of type 2 diabetes mellitus (DM) have increased over the past two decades and caused much health burden across the world (1, 2). Past studies have demonstrated that type 2 DM is associated with an elevated risk of stroke (3, 4). Stroke in patients with type 2 DM has a poor prognosis, which is marked by worse mortality outcomes relative to that in several other diabetes-related comorbidities, including coronary heart diseases (4). It affects approximately 40% of patients with ischemic stroke who had been diagnosed with diabetes in the United States (5). A study reported that controlling glucose levels with intensive diabetes therapy could reduce the risk of stroke by 57% (6).

Sodium-glucose co-transporter-2 (SGLT2) inhibitors are used in patients with type 2 DM as glucose-lowering therapies targeting SGLT2 (7, 8). Although these drugs are primarily indicated for diabetes, several studies have examined their use in the primary and secondary prevention of stroke (9, 10). Animal studies have demonstrated a neuroprotective effect of SGLT2 inhibitors, which play an important role in antioxidant, anti-inflammatory, and anti-apoptotic mechanisms (11–13). SGLT2 inhibitors also improve the endothelial function, prevent remodeling, and exert a protective effect on the neurovascular unit and the blood–brain barrier, which can be promising in stroke therapy (14). However, the results of previous studies are inconsistent in a clinical setting (15–17). Therefore, the objective of the present study was to evaluate the risk of new-onset stroke (NOS) associated with the prescription of SGLT2 inhibitors in a nationwide cohort study of patients with type 2 DM in Taiwan.

## Materials and methods

### Study design

This is a retrospective study conducted on a population-based cohort using data from the insurance claims provided by the Taiwanese Bureau of National Health Insurance (TBNHI) from January 2004 to December 2019. This database contains anonymized longitudinal medical records that store the claims' information forms in two tables: a visit table and a prescription table. The visit tables contain the patient's identification numbers, sex, age, three diagnostic codes for outpatient and five for inpatient visits, medications, drug doses, medical expenditures, and hospital and physician information. The prescription table contains the quantity and expenditure for all administered drugs, operations, and treatments undertaken.

Patients included in this study were of age at least 20 years, with a newly diagnosed case of type 2 DM with or without prescribed SGLT2 inhibitors between May 2016 and December 2019. SGLT2-inhibitor users were defined as patients who received at least an SGLT2 inhibitor prescription for 180 days during the study period. In contrast, non-SGLT2 inhibitor users were patients who did not receive an SGLT2 inhibitor prescription throughout the study period.

### Study population

**Figure 1:**
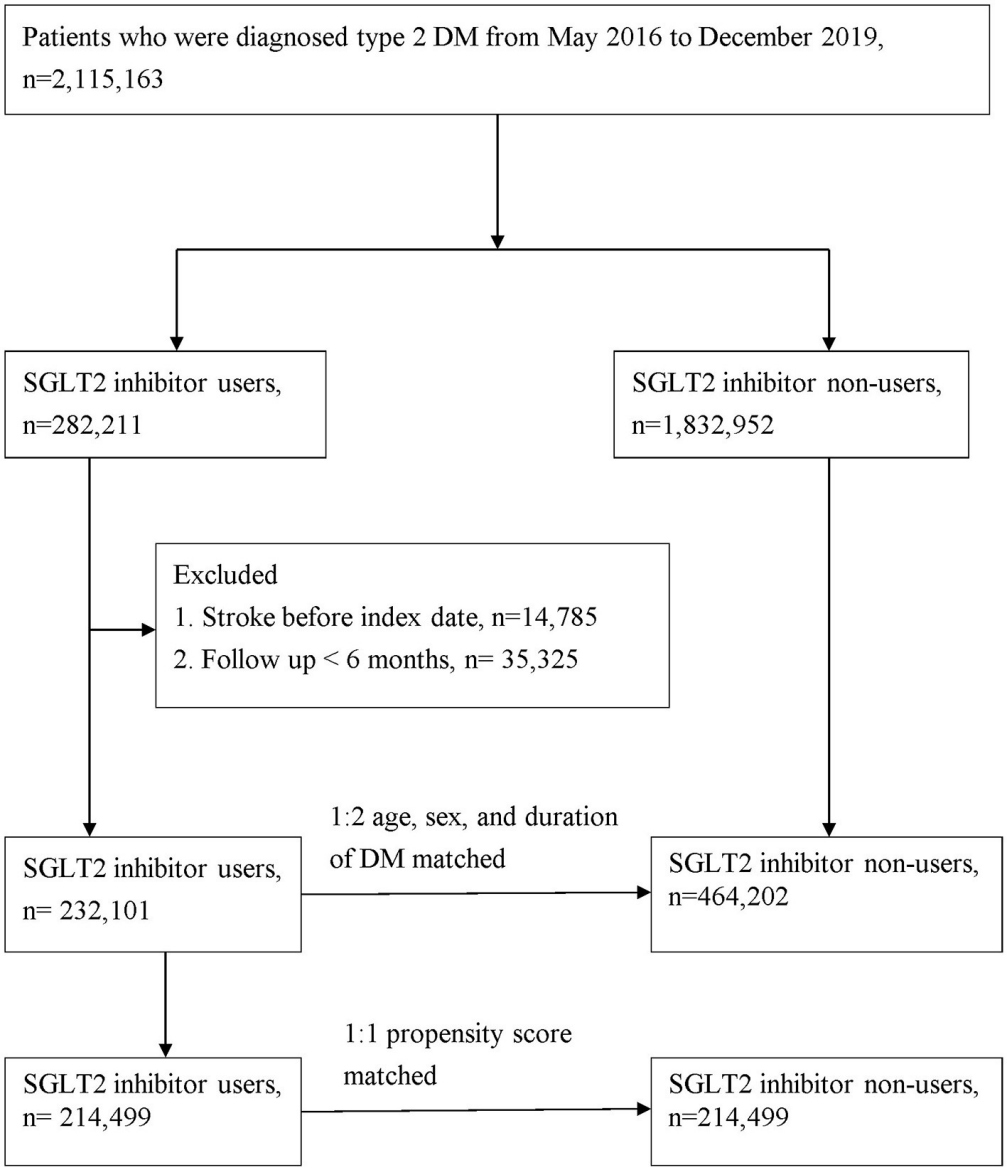
Patient flow chart.

The study population comprised patients with type 2 DM (ICD-10-CM, E11) who were admitted to the hospital or visited the hospital as an outpatient between May 1, 2016 and December 31, 2019. At least one of the following enrollment criteria was required to be met for inclusion in this study: (1) two or more outpatient visits within 6 months, (2) all antidiabetic drugs were continuously prescribed to the patients for >6 months during the follow-up period, or (3) one or more inpatient admissions with a diagnosis of type 2 DM. The primary endpoint was the development of stroke, which was defined by the time a stroke (ICD-10-CM codes I60, I61, I62, I63, I65, I66, I67.84, G45, G46) code first appeared in the inpatient or outpatient claim records. Comorbidities related to stroke were defined according to the ICD-10-CM code and included coronary heart disease (ICD-10-CM code I20–I25), hypertension (ICD-10-CM code I10), hyperlipidemia (ICD-9-CM code E78.1–E78.5), chronic kidney disease (ICD-10-CM code N18), chronic liver disease (ICD-10-CM code K71, K75, K76), chronic obstructive pulmonary disease (ICD-10-CM code J44), atrial fibrillation and flutter (ICD-10-CM code I48), and rheumatoid arthritis (ICD-9-CM code M05). Patients who fulfilled any of the following criteria were excluded from the study: (1) prior history of stroke before May 1, 2016 and (2) patient age of <20 years. Considering the differences in the baseline characteristics and stroke risk between the SGLT2-inhibitor users and non-SGLT2-inhibitor users, we applied age-, sex-, and type 2 DM duration matching at a ratio of 1:2 for patients with type 2 DM with and without SGLT2 inhibitor use. Finally, the study group comprised 232,101 participants with type 2 DM who were SGLT2 inhibitor users, and the control group included 464,202 randomly selected participants with type 2 DM who were non-SGLT2-inhibitor users (Figure 1). We also conducted propensity score matching with age, sex, duration of type 2 DM, comorbidities, and drug index date at a ratio of 1:1 for sensitivity analysis in patients with type 2 DM with and without the use of an SGLT2 inhibitor (Figure 1).

### Statistical analysis

Data were presented as valid percentages and the mean values with a standard deviation. Differences in the demographic data and clinical characteristics between SGLT2-inhibitor users and non-SGLT2-inhibitor users were examined using a *t*-test for continuous variables, whereas Chi-square tests were performed for categorical variables. The Cox proportional hazard regression model was applied to compare the risk of developing study events between the SGLT2 inhibitor group and the non-SGLT2 inhibitor group. Adjusted hazard ratios (HRs) and 95% confidence intervals (CIs) were calculated after adjusting for important risk factors toward developing the study events, including age, sex, concurrent medication, and comorbidities. The risk of study outcomes over time for the SGLT2 inhibitor group compared with the non-SGLT2 inhibitor group was determined by survival analysis using the Kaplan–Meier method.

We also conducted a sensitivity analysis to test the robustness of our primary findings. Initially, a propensity score was calculated for each patient to minimize confounding by indication, when patients with other risk factors between the SGLT2 inhibitor user group and non-SGLT2 inhibitor user group. Then, the propensity score matching (1:1) and absolute standardized difference (ASD) were performed to estimate the difference between the two groups. An ASD of <0.10 implied a negligible difference in the potential confounders between the two groups.

In addition, we conducted subgroup analyses stratified by sex, age, duration of type 2 DM, presence of comorbidities, and concurrent medication at baseline for the primary outcomes of NOS. Statistical significance was considered at *P* < 0.05. All statistical calculations were performed using the statistical analysis software, version 9.3 (SAS Institute, Inc., Cary, NC, USA).

## Results

### Study population and baseline characteristics

A total of 696,303 patients were enrolled in the present study, with the SGLT2-inhibitor user group consisting of 232,101 individuals from the NHIRD who were diagnosed with type 2 DM from May 2016 through December 2019. This group was compared with 464,202 control patients who were non-SGLT2-inhibitor users at a 1:2 ratio (Figure 1). There were more men (55.94%) than women (44.06%) in this study. At the baseline, patients receiving SGLT2 inhibitor had more comorbidities, except for rheumatoid arthritis, and they used more concurrent medication than those not receiving SGLT2 inhibitor (Table 1).

**Table 1:** Baseline characteristics of all patients.

		**2:1 sex, age matching**			**1:1 Propensity score matching**		
**Characteristics**		**Non- SGLT2** **(*n* = 464,202)**	**SGLT2** **(*n* = 232,101)**	***P***	**Non-SGLT2** **(*n* = 214,499)**	**SGLT2** **(*n* = 214,499)**	**ASD**
Sex				1.0000			0.00177
Female		204,534 (44.06%)	102,267 (44.06%)		94,518 (44.06%)	94,707 (44.15%)	
Male		259,668 (55.94%)	129,834 (55.94%)		119,981 (55.94%)	119,792 (55.85%)	
Age				1.0000			0.00000
<50		114,804 (24.73%)	57,402 (24.73%)		53,466 (24.93%)	53,131 (24.77%)	
51–60		138,494 (29.83%)	69,247 (29.83%)		64,132 (29.90%)	64,128 (29.90%)	
61–70		142,538 (30.71%)	71,269 (30.71%)		66,008 (30.77%)	65,896 (30.72%)	
>70		68,366 (14.73%)	34,183 (14.73%)		30,893 (14.4%)	31,344 (14.61%)	
Years (Mean ± SD)		58.34 ± 12.21	58.34 ± 12.21	1.0000	58.44 ± 11.89	58.29 ± 12.23	
DM history				<0.0001			0.02967
< =2 years		133,455 (28.75%)	59,608 (25.68%)		54,688 (25.50%)	55,752 (25.99%)	
3-4 years		243,394 (52.43%)	126,088 (54.32%)		115,391 (53.80%)	115,875 (54.02%)	
≥5 years		87,353 (18.82%)	46,405 (19.99%)		44,420 (20.71%)	42,872 (19.99%)	
**Comorbidities**							
dv11	Hypertension	250,659 (54%)	139,336 (60.03%)	<0.0001	128,819 (60.06%)	12,738 5(59.39%)	0.01363
dv13	Coronary artery disease	51,129 (11.01%)	41,448 (17.86%)	<0.0001	33,966 (15.84%)	35,030 (16.33%)	0.01350
dv14	Hyperlipidemia	257,784 (55.53%)	153,956 (66.33%)	<0.0001	142,463 (66.42%)	140,575 (65.54%)	0.01858
dv19	Chronic kidney disease	104,962 (22.61%)	59,599 (25.68%)	<0.0001	57,593 (26.85%)	54,907 (25.60%)	0.02847
dv20	Chronic liver disease	50,928 (10.97%)	26,537 (11.43%)	<0.0001	24,725 (11.53%)	24,501 (11.42%)	0.00328
dv66	COPD	15,910 (3.43%)	8,446 (3.64%)	<0.0001	7,301 (3.40%)	7,631 (3.56%)	0.00839
dv29	Atrial fibrillation and flutter	4,902 (1.06%)	3,824 (1.65%)	<0.0001	3,087 (1.44%)	3,149 (1.47%)	0.00242
	Rheumatoid arthritis	3,188 (0.69%)	1,285 (0.55%)	0.01696	1,168 (0.54%)	1,202 (0.56%)	0.00214
**Concurrent medication**							
Dr1	NSAIDs	263,337 (56.73%)	133,108 (57.35%)	<0.0001	122,355 (57.04%)	122,768 (57.23%)	0.00389
Dr2	Corticosteroids	88,850 (19.14%)	45,398 (19.56%)	<0.0001	41,286 (19.25%)	41,608 (19.40%)	0.00380
Dr3	PPIs	35,647 (7.68%)	18,410 (7.93%)	0.0002	16,619 (7.75%)	16,739 (7.80%)	0.00209
Dr4	H2-receptor antagonists	120,629 (25.99%)	61,091 (26.32%)	0.0027	55,435 (25.84%)	56,109 (26.16%)	0.00716
Dr5	Aspirins	92,245 (19.87%)	63,518 (27.37%)	<0.0001	55,176 (25.72%)	55,748 (25.99%)	0.00609
Dr25	Statins	240,244 (51.75%)	162,084 (69.83%)	<0.0001	147,212 (68.63%)	146,131 (68.13%)	0.01084
Dr13	Biguanides	242,784 (52.3%)	151,068 (65.09%)	<0.0001	134,691 (62.79%)	136,345 (63.56%)	0.01599
Dr14	Sulfonylureas	155,979 (33.6%)	101,140 (43.58%)	<0.0001	91,743 (42.77%)	90,022 (41.97%)	0.01624
Dr15	Alpha glucosidase inhibitors	45,540 (9.81%)	43,008 (18.53%)	<0.0001	34,432 (16.05%)	35,391 (16.50%)	0.01211
Dr16	Thiazolidinediones	43,754 (9.43%)	41,938 (18.07%)	<0.0001	34,607 (16.13%)	34,857 (16.25%)	0.00316
Dr17	DPP4 inhibitors	99,152 (21.36%)	93,734 (40.39%)	<0.0001	80,445 (37.50%)	79,384 (37.01%)	0.01023
Dr18	Insulins	71,925 (15.49%)	57,020 (24.57%)	<0.0001	48,358 (22.54%)	48,840 (22.77%)	0.00537
Dr26	GLP-1 receptor agonists	5,101 (1.1%)	4,244 (1.83%)	<0.0001	3,763 (1.75%)	3,665 (1.71%)	0.00350

COPD, chronic obstructive pulmonary disease; DPP4, Dipeptidyl peptidase 4; GLP-1, Glucagon-like peptide-1; NSAID, Non-steroid anti-inflammatory drug; PPI, proton pump inhibitor; ASD, absolute standardized difference; PSM, propensity score matching; SD, standard deviation.

### Analysis of the main TBNHI cohort

**Figure 2:**
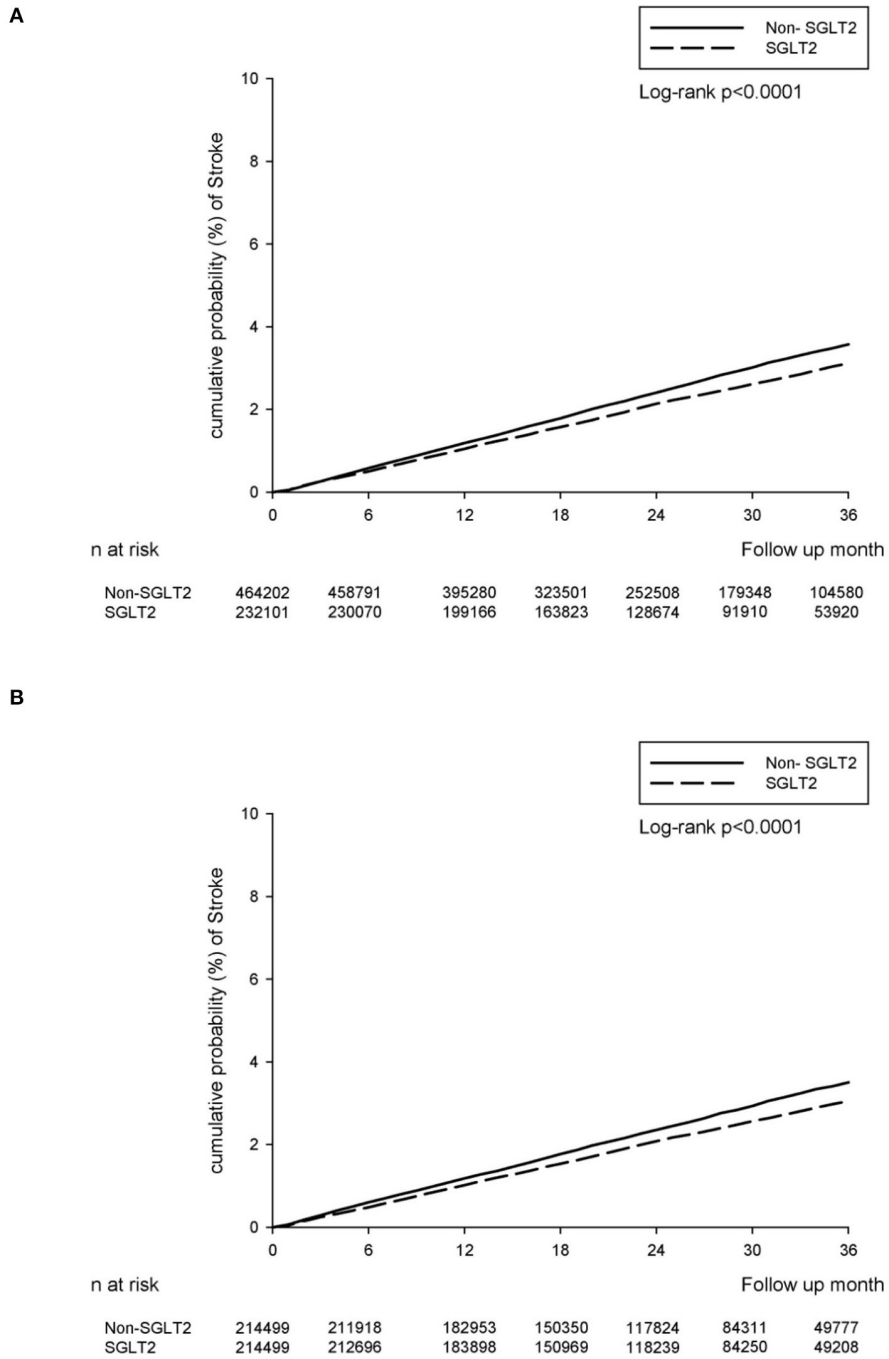
The cumulative incidence rate of developing stroke between SGLT2-inhibitor group and non-SGLT2-inhibitor group. **(A)** The main TBNHI Cohort. **(B)** The propensity score-matched cohort.

During the follow-up, 5,186 and 11,701 NOSs events were recorded in the SGLT2-inhibitor user and non-SGLT2-inhibitor user groups, respectively. The event rate was 9.20 per 10 000 person-months (95% CI 8.95–9.45) for SGLT2-inhibitor users when compared with 10.50 (95% CI 10.30–10.60) for non-SGLT2-inhibitor users. There was a significantly lower the incidence rate of NOS after adjusting for the duration of type 2 DM history, sex, age, comorbidities, and concurrent medication among the SGLT2-inhibitor users when compared to that among the non-SGLT2-inhibitor users (adjusted HR: 0.85; 95% CI: 0.82–0.88) (Table 2). The cumulative incidence rate of developing stroke was also lower in the SGLT2-inhibitor users than in the non-SGLT2-inhibitor in the Kaplan–Meier survival analysis (*P* < 0.0001; Figure 2A).

**Table 2:** Incidence rate of stroke.

	**2:1 sex age matching**		**1:1 Propensity score matching**	
	**Non- SGLT2**	**SGLT2**	**Non- SGLT2**	**SGLT2**
*N*	464,202	232,101	214,499	214,499
Follow up person months	11,135,130	5,634,359	5,177,840	5,191,193
New case	11,701	5,186	5,328	4,678
Incidence rate^*^(95% C.I.)	10.50 (10.30–10.60)	9.20 (8.95–9.45)	10.20 (10.00–10.50)	9.01 (8.75–9.27)
Crude Relative risk (95% C.I.)	Reference	0.88 (0.85–0.91)	Reference	0.88 (0.84–0.91)
Adjusted HR^*^ (95% C.I.)^†^	Reference	0.85 (0.82–0.88)	Reference	0.87 (0.84–0.91)

* Incidence rate, per 10,000 person-months.

† adjusted hazard ratio, the covariates including duration of DM history, sex, age, co-morbidities, and medication at baseline.

### Propensity score-matched analysis

We included 428,998 patients (214,499 in the SGLT2-inhibitor group and 214,499 in the non-SGLT2-inhibitor group) in the propensity score matching, and the baseline characteristics of sex, age, and duration of type 2 DM did not differ (Table 1). At the baseline, the non-SGLT2-inhibitor group had more comorbidities, except for coronary artery disease, chronic obstructive pulmonary disease, atrial fibrillation and flutter, and rheumatoid arthritis than the SGLT2-inhibitor group. However, the SGLT2 inhibitor users used more concurrent medication, except statins, sulfonylureas, dipeptidyl peptidase 4 inhibitors, and glucagon-like peptide-1 receptor agonists than the non-SGLT2 inhibitor users (Table 1).

There were 4,678 and 5,328 NOS events recorded in the SGLT2-inhibitor and non-SGLT2-inhibitor groups, respectively, in the follow-up period. The event rate was 9.01 per 10 000 person-months (95% CI 8.75–9.27) for the SGLT2-inhibitor group compared with 10.20 (95% CI 10.00–10.50) for the non-SGLT2-inhibitor group. The relative risk of NOS after adjusting the duration of type 2 DM history, sex, age, comorbidities, and concurrent medication demonstrated a decreasing risk of incident stroke in the SGLT2 inhibitor group when compared to those in the non-SGLT2-inhibitor group (adjusted HR: 0.87; 95% CI: 0.84–0.91) (Table 2). Similarly, the SGLT2-inhibitor group revealed a significantly lower cumulative incidence rate of developing stroke than the non-SGLT2-inhibitor group as per the Kaplan–Meier survival analysis (*P* < 0.0001, Figure 2B).

### Subgroup analysis

The results of the subgroup analyses revealed that, after adjusting for the duration of type 2 DM history, sex, age, comorbidities, and concurrent medication were partly consistent with the results of the main analyses (Table 3). The two groups were different in terms of their incidental stroke, with the SGLT2 inhibitor users exhibiting a substantially high risk of NOS with male, an adjusted HR = 1.34 (95% CI: 1.30 to 1.39) than female. Compared with younger patients (aged < 50), elderly patients exhibited a significantly higher risk of NOS (aHR 1.59, 95% CI 1.51–1.68 for patients aged 50–60; aHR 2.24, 95% CI 2.13–2.36 for patients aged 60–70; aHR 3.67, 95% CI 3.48–3.88 for patients aged > 70). The duration of type 2 DM history were higher in the < =2 or 2–4 years than in the ≥4 years. Patients with hypertension, chronic kidney disease, chronic obstructive pulmonary disease, atrial fibrillation and flutter, and rheumatoid arthritis were also at significantly higher risks of NOS (aHR = 1.22, 1.17, 1.08, 1.79, and 1.23, respectively). However, patients with hyperlipidemia and chronic liver disease have significantly lower risks of NOS (aHR = 0.77, and 0.81, respectively). Similar findings were also noted for concurrent medication of statins (aHR 0.84, 95% CI 0.81–0.86 in the main TBNHI cohort; aHR 0.88, 95% CI 0.84–0.92 in the propensity score matching), biguanides (aHR 0.77, 95% CI 0.75–0.79 in the main TBNHI cohort; aHR 0.85, 95% CI 0.82–0.89 in the propensity score matching), thiazolidinediones (aHR 0.89, 95% CI 0.85–0.93 in the main TBNHI cohort; aHR 0.93, 95% CI 0.88–0.98 in the propensity score matching), and glucagon-like peptide-1 receptor agonists (aHR 0.84, 95% CI 0.71–0.98 in the main TBNHI cohort; aHR 0.77, 95% CI 0.63–0.93 in the propensity score matching). However, an increased risk of NOS was noted for concurrent medication with non-steroid anti-inflammatory drugs (aHR 1.01, 95% CI 0.98–1.05 in the main TBNHI cohort; aHR 1.05, 95% CI 1.01–1.05 in the propensity score matching), corticosteroids (aHR 1.07, 95% CI 1.03–1.11 in the main TBNHI cohort; aHR 1.08, 95% CI 1.02–1.13 in the propensity score matching), proton pump inhibitors (aHR 1.19, 95% CI 1.13–1.25 in the main TBNHI cohort; aHR 1.20, 95% CI 1.12–1.20 in the propensity score matching), H2-receptor antagonists (aHR 1.05, 95% CI 1.02–1.09 in the main TBNHI cohort; aHR 1.07, 95% CI 1.02–1.12 in the propensity score matching), aspirins (aHR 1.53, 95% CI 1.48–1.59 in the main TBNHI cohort; aHR 1.55, 95% CI 1.49–1.62 in the propensity score matching), sulfonylureas (aHR 1.09, 95% CI 1.06–1.13 in the main TBNHI cohort; aHR 1.14, 95% CI 1.10–1.19 in the propensity score matching), alpha-glucosidase inhibitors (aHR 1.03, 95% CI 0.98–1.07 in the main TBNHI cohort; aHR 1.06, 95% CI 1.01–1.12 in the propensity score matching), Dipeptidyl peptidase 4 inhibitors (aHR 1.05, 95% CI 1.02–1.09 in the main TBNHI cohort; aHR 1.08, 95% CI 1.03–1.12 in the propensity score matching), and insulins (aHR 1.62, 95% CI 1.56–1.68 in the main TBNHI cohort; aHR 1.67, 95% CI 1.60–1.74 in the propensity score matching) (Table 3).

**Table 3:** Multiple Cox regression to estimate the hazard ratio for subgroup analysis.

	**aHR (95% CI)**	
	**2:1 sex, age matching**	**1:1 propensity score matching**
Sex		
Female	reference	reference
Male	1.34(1.30–1.39)	1.33(1.27–1.38)
Age		
<50	reference	reference
51–60	1.59(1.51–1.68)	1.51(1.41–1.63)
61–70	2.24(2.13–2.36)	2.17(2.02–2.32)
>70	3.67(3.48–3.88)	3.55(3.31–3.82)
Duration of type 2 DM history		
< =2 years	1.21(1.14–1.28)	1.27(1.11–1.37)
2–4 years	1.16(1.11–1.23)	1.20(1.12–1.28)
>=4 years	reference	reference
Comorbidity(ref: non-comorbidity)		
Hypertension	1.22(1.18–1.26)	1.28(1.23–1.34)
Coronary artery disease	1.02(0.97–1.06)	1.02(0.97–1.07)
Hyperlipidemia	0.77(0.74–0.79)	0.80(0.77–0.83)
Chronic kidney disease	1.17(1.13–1.21)	1.16(1.11–1.21)
Chronic liver disease	0.81(0.77–0.85)	0.79(0.74–0.85)
Malignancy	1.02(0.96–1.08)	1.03(0.95–1.13)
COPD	1.08(1.01–1.16)	1.06(0.97–1.15)
Atrial fibrillation and flutter	1.79(1.64–1.95)	1.82(1.64–2.02)
Rheumatoid Arthritis	1.23(1.04–1.44)	1.15(0.91–1.45)
Medication (reference: non-medication)		
NSAIDs	1.00(0.97–1.04)	1.05(1.01–1.09)
Corticosteroids	1.07(1.03–1.11)	1.08(1.02–1.13)
PPIs	1.19(1.13–1.25)	1.20(1.12–1.28)
H2-receptor antagonists	1.05(1.02–1.09)	1.07(1.02–1.12)
Aspirins	1.53(1.48–1.59)	1.55(1.49–1.62)
Statins	0.84(0.81–0.86)	0.88(0.84–0.92)
Biguanides	0.77(0.75–0.79)	0.85(0.82–0.89)
Sulfonylureas	1.09(1.06–1.13)	1.14(1.10–1.19)
Alpha glucosidase inhibitors	1.03(0.98–1.07)	1.06(1.01–1.12)
Thiazolidinediones	0.89(0.85–0.93)	0.93(0.88–0.98)
DPP4 inhibitors	1.05(1.02–1.09)	1.08(1.03–1.12)
Insulins	1.62(1.56–1.68)	1.67(1.60–1.74)
GLP-1 receptor agonists	0.84(0.71–0.98)	0.77(0.63-y0.93)

COPD, chronic obstructive pulmonary disease; DM, diabetes mellitus; DPP4, Dipeptidyl peptidase 4; GLP-1, Glucagon-like peptide-1; NSAID, Non-steroid anti-inflammatory drug; PPI, proton pump inhibitor.

## Discussion

The present findings suggest that the incidence of NOS was decreased in type 2 DM patients who were SGLT2-inhibitor users compared with those who were not. Sensitivity analysis was also consistent with the main analysis. The subgroups analysis identified the concurrent use of statins, biguanides, thiazolidinediones, and glucagon-like peptide-1 receptor agonists as having a protective effect against developing NOS. However, we observed the increased risk based on whether non-steroid anti-inflammatory drugs, corticosteroids, proton pump inhibitors, H2-receptor antagonists, aspirins, sulfonylureas, alpha-glucosidase inhibitors, dipeptidyl peptidase 4 inhibitors, and insulins were prescribed for concurrent use with an SGLT2 inhibitor.

Hypertension, type 2 DM, and obesity are identified as the most important risk factors for stroke (18). Several experimental studies reported improvements in these risk factors in diabetic and obese or stroke-prone mice and rats after treatment with SGLT2 inhibitors (11–13, 19). *In vitro* data has shown that the SGLT2 inhibitor significantly increased survival (67%) of spontaneously hypertensive stroke-prone rats when compared with controls (13). The authors observed that SGLT2 inhibitor-treated rats had weight and blood pressure reduction, which could explain the reduced stroke risk and increased survival. However, the effects of SGLT2 inhibitors on stroke prevention were contradictory in different clinical trials. In the Empagliflozin Cardiovascular Outcomes and Mortality in Type 2 Diabetes (EMPA-REG OUTCOME) trial (17), empagliflozin users were found to be associated with an insignificantly increased risk of stroke when compared to empagliflozin non-users (HR, 1.18; 95% CI, 0.89–1.56; *P* = 0.26). On the other hand, canagliflozin users were found to be associated with an insignificantly decreased risk of stroke relative to canagliflozin non-users (HR, 0.87; 95% CI, 0.69–1.09) in the Cardiovascular and Renal Events in Type 2 Diabetes (CANVAS) trial (20). However, several meta-analyses have demonstrated that SGLT2 inhibitors may lower the risk of embolic stroke (9, 21, 22). Their results were the same as ours and they suggested a possible protective effect of SGLT2 inhibitors including different populations and the level of renal functions.

In our study, subgroups analyses demonstrated that the patients' concurrent use of statins, biguanides, thiazolidinediones, and glucagon-like peptide-1 receptor agonists had a protective effect against developing NOS, whereas patients' concurrent use of non-steroid anti-inflammatory drugs, corticosteroids, proton pump inhibitors, H2-receptor antagonists, aspirins, sulfonylureas, alpha-glucosidase inhibitors, dipeptidyl peptidase 4 inhibitors, and insulins showed an increased risk of developing NOS. This result demonstrates that different drugs may play a major role in lowering or increasing the risk of NOS when combined with SGLT2 inhibitors for patients with type 2 DM, which conforms to previous reports (23–27).

Other than antidiabetic effects, SGLT2 inhibitors also promoted natriuresis and osmotic diuresis to lower blood pressure in patients with cardiovascular disease and heart failure (28–30). As evidence of the efficacy of SGLT-2 inhibitors continued to grow, many trails and meta-analysis on these drugs have expanded their prescriptions from diabetes patients only to also include patients with HF without type 2 DM (28–32). Furthermore, the safety and dose-response relationship of SGLT2 inhibitors were recommended in the clinical practice (33–35).

In summary, there is negative association between the use of SGLT2 inhibitors and the risk of NOS in patients with type 2 DM. The decreased risk of NOS in patients with type 2 DM was greater among patients with concurrent use of statins, biguanides, thiazolidinediones, and glucagon-like peptide-1 receptor agonists. Therefore, we suggest that the long-term use of SGLT2 inhibitors may help reduce the incidence of NOS in patients with type 2 DM.

## Data availability statement

The raw data supporting the conclusions of this article will be made available by the authors, without undue reservation.

## Ethics statement

This study was approved by the Ethics Committee of the Chung Shan Medical University Hospital (CS1-21037). Written informed consent for participation was not required for this study in accordance with the national legislation and the institutional requirements.

## Author contributions

T-KL and M-CC: conceptualization, methodology, formal analysis, and writing–original draft. Y-HC and J-YH: formal analysis and validation. P-LL: formal analysis. T-KL, Y-HC, J-YH, and M-CC: data curation. L-FP and G-PJ: conceptualization, investigation, writing–review and editing, supervision, project administration, and funding acquisition. All authors read the study and approved the manuscript for publication.

## Funding

This study was supported by grants (CSH-2021-C-001) from Chung Shan Medical University Hospital.

## Conflict of interest

The authors declare that the research was conducted in the absence of any commercial or financial relationships that could be construed as a potential conflict of interest.

## Publisher's note

All claims expressed in this article are solely those of the authors and do not necessarily represent those of their affiliated organizations, or those of the publisher, the editors and the reviewers. Any product that may be evaluated in this article, or claim that may be made by its manufacturer, is not guaranteed or endorsed by the publisher.
